# The PPARγ agonist pioglitazone prevents TGF-β induced renal fibrosis by repressing EGR-1 and STAT3

**DOI:** 10.1186/s12882-019-1431-x

**Published:** 2019-07-05

**Authors:** Ágnes Németh, Miklós M. Mózes, Laurent Calvier, Georg Hansmann, Gábor Kökény

**Affiliations:** 1 Department of Pathophysiology, Semmelweis University, Nagyvárad tér 4, Budapest, H-1089 Hungary; 2 Department of Pediatric Cardiology and Critical Care, Hannover Medical School, Hannover, Germany

**Keywords:** Kidney, Renal fibrosis, PPARγ, TGF-β, Transcription factors

## Abstract

**Background:**

It has been proposed that peroxisome proliferator-activated receptor-γ (PPARγ) agonists might reduce renal fibrosis, however, several studies had contradictory results. Moreover, the possible interaction of TGF-β_1_, PPARγ, and transcription factors in renal fibrosis have not been investigated. We hypothesized that oral pioglitazone treatment would inhibit TGF-β–driven renal fibrosis and its progression, by modulating profibrotic transcription factors in TGF-β_1_ transgenic mice.

**Methods:**

Male C57Bl/6 J mice (control, CTL, *n* = 14) and TGF-β overexpressing transgenic mice (TGFβ, *n* = 14, having elevated plasma TGF-β_1_ level) were divided in two sets at 10 weeks of age. Mice in the first set were fed with regular rodent chow (CTL and TGFβ, *n* = 7/group). Mice in the second set were fed with chow containing pioglitazone (at a dose of 20 mg/kg/day, CTL + Pio and TGFβ+Pio, *n* = 7/group). After 5 weeks of treatment, blood pressure was assessed and urine samples were collected, and the kidneys were analyzed for histology, mRNA and protein expression.

**Results:**

TGF-β_1_ induced glomerulosclerosis and tubulointerstitial damage were significantly reduced by pioglitazone. Pioglitazone inhibited renal mRNA expression of all the profibrotic effectors: type-III collagen, TGF-β_1_, CTGF and TIMP-1, and alike transcription factors cFos/cJun and protein expression of EGR-1, and STAT3 protein phosphorylation.

**Conclusions:**

Oral administration of PPARγ agonist pioglitazone significantly reduces TGF-β_1_-driven renal fibrosis, via the attenuation of EGR-1, STAT3 and AP-1. This implies that PPARγ agonists might be effective in the treatment of chronic kidney disease patients.

**Electronic supplementary material:**

The online version of this article (10.1186/s12882-019-1431-x) contains supplementary material, which is available to authorized users.

## Background

Renal fibrosis is the hallmark of several chronic kidney diseases (diabetes mellitus, arterial hypertension) that progress to end-stage renal disease (ESRD), and thus produce a serious health burden worldwide [1]. The dynamic balance of extracellular matrix protein (ECM) synthesis and degradation is mainly regulated by matrix metalloproteinases (MMPs) and their inhibitors, the tissue inhibitors of metalloproteinases (TIMPs). In fibrosis that occurs in multiple organs, this MMP/TIMP balance is disrupted. Transforming growth factor-β_1_ (TGF-β_1_) is a multifunctional cytokine involved in various pathologic conditions, including carcinogenesis, and tissue fibrosis [2, 3]. TGF-β_1_ signaling can induce TIMP-1 transcription at promoter level via activator protein-1 complex (AP-1) [4]. TGF-β_1_ transgenic mice with elevated plasma levels of TGF-β_1_ develop severe renal fibrosis, underscoring the central role of TGF-β_1_ in the pathogenesis of fibrotic kidney diseases [5].

Among the several profibrotic transcription factors, early growth response factor-1 (EGR-1) contributes to fibrosis by directly stimulating collagen synthesis [6] and myofibroblast differentiation [7]. The Janus kinase family (JAK) and the signal transducers and activators of transcription (STATs) signaling pathways also play important role in several models of renal diseases [8–10]. STAT3 influences cell proliferation [11–13] and fibrosis [14] by inducing the expression of multiple genes, including TGF-β_1_ [15]. Conversely, we identified a novel non-canonical, proliferative TGF-β_1_-pSTAT3-pFoxO1 signaling axis in vascular smooth muscle cells [16].

Peroxisome proliferator-activated receptor-γ (PPARγ) is an ubiquitary, anti-fibrotic and vasoprotective nuclear hormone receptor and transcription factor. PPARγ agonists (e.g. pioglitazone) have been widely used in the treatment of diabetes mellitus [17] and the prevention of macrovascular events and the development of diabetes in patients with insulin resistance [18].

Moreover, PPARγ agonists have been also shown to reduce renal damage in animal models of ischemia reperfusion injury, autosomal dominant polycystic kidney disease (ADPKD) or nondiabetic chronic kidney diseases [19]. Interestingly, despite a beneficial effect of the (otherwise liver-toxic) PPARγ agonist troglitazone was reported in the unilateral ureter obstruction (UUO) model of renal fibrosis [20], others could not confirm these results using pioglitazone [21]. In addition to these conflicting results, the possible interaction of PPARγ, TGF-β_1_ and EGR-1 in renal fibrosis has not been investigated.

Here, we aimed to study whether the chronic administration of PPARγ agonist pioglitazone could influence renal EGR-1, STAT3 and AP-1 expression, and ameliorate renal fibrosis in TGF-β_1_ overexpressing mice. We report that oral administration of pioglitazone effectively reduced glomerulosclerosis, tubular injury and interstitial fibrosis by inhibiting EGR-1 and TIMP-1 expression. Our results might help to clarify the beneficial role of PPARγ in preventing or reversing TGFβ_1_-driven renal fibrosis.

## Methods

### Animals and experimental design

Male C57Bl6/J (B6) and B6-Alb/TGF-β_1_(cys^223,225^ser) [5] (TGF-β1 transgenic mice with elevated circulating TGF-β_1_ level) were housed at the Semmelweis University NET GMO facility under standard specific pathogen free (SPF) conditions, with a 12/12 h light/dark cycle. In TGF-β1 transgenic mice, the transgene construct consists of the full-length porcine TGF-β1 cDNA (with mutated cysteine 223 and 225 to serine which leads to production of active TGF-β1) driven by the albumin promoter and enhancer, and the transgene was incorporated into the Y chromosome therefore only male mice are transgenic. The expression of the transgene starts after birth in the hepatocytes, and leads to constitutive secretion of active TGF-β1 into the bloodstream. The mouse strains were bred and maintained at the NET GMO SPF facility. The animals had free access to standard rodent chow (Altromin 1314 TPF) and filtered drinking water. 10 week old male C57Bl/6 J control (CTL) and TGF-β_1_ transgenic mice (TGFβ) were randomly divided in two sets. The number of animals was calculated by the Ethical Committee to provide the minimum sample size needed for relevant results. The first set of mice received regular chow. The second set of mice were treated orally with pioglitazone (20 mg/kg/day) for 5 weeks (CTL + Pio and TGFβ+Pio). Pioglitazone was incorporated in the chow, and average food consumption was assessed at the beginning of the study. Sample size was *n* = 7/group. At 15 weeks of age, urine spot samples were obtained by sterile punction of the bladder under isoflurane (2%) anesthesia, blood pressure was measured and mice were perfused by intracardiac cannula under narcosis for 20 min with 4 °C physiological saline. Then, mice were euthanized with 5% isoflurane and kidneys were harvested for histology and mRNA expression analyses. All assays were performed in duplicates for each sample to ensure reliability of single values.

### Animal genotyping

The presence of TGF-β_1_ transgene was verified from genomic DNA (1–2 mm tail samples) by polymerase chain reaction (PCR). DNA was extracted by Tris-NaOH method. Briefly, samples were incubated in 200 μl of 0.1 N NaOH in a microcentrifuge tube at 96 °C for 10 min, mixed and cooled on ice. After briefly spun, 50 μl Tris (pH 8) was added to the lysates, mixed and centrifuged at 20000 g for 6 min at 4 °C. Supernatants were pipetted into a new tube, and 2.5 μl of each supernatant was used for the PCR.

The following Alb/TGFb primer sequences were used: sense: 5′-GGCAAACATACGCAAGGGA-3′; antisense: 5′-AGAATCTGGCCGCGAATGG-3′. The PCR reactions were the following: initial denaturation at 95 °C for 2 min, then 35 cycles of 95 °C for 50 s, 65 °C for 50 s and 72 °C for 75 s. The PCR products were separated on 1.5% agarose gels to detect the 370 base pair product of the transgene [22].

### Measurement of blood pressure

Systemic blood pressure was measured invasively under isoflurane analgesia, followed by organ harvest. A 1.4F microtip catheter (Millar Instruments, USA) inserted into the right carotid artery/ aortic arch where the systemic blood pressure was measured. Blood pressure curves were recorded and analyzed using a PowerLab recording unit and LabChart software (AD Instruments, Colorado Springs, USA).

### Determination of plasma TGF-β_1_ levels

During harvest, blood samples from the aorta were harvested in siliconized microcentrifuge tubes containing EDTA as anticoagulant (100 μl/mouse). Blood samples were centrifuged at 1000 g for 20 min and the isolated plasma was centrifuged again in new siliconized tubes at 10000 g for 10 min. Supernatants (10 ul) were used to determine plasma TGF-β_1_ level using a commercial ELISA kit (R&D Quantikine TGF-β_1_ ELISA, Minneapolis, USA).

### Determination of proteinuria

The amount of excreted protein (BCA assay, Thermo Fisher, Waltham, MA, USA) and creatinine (Creatinine Kit, Diagnosticum ZRt, Budapest, Hungary) were measured from spot urine samples according to the manufacturer’s protocols. Proteinuria was expressed as urinary protein to creatinine ratio (UPCR), in order to normalize for GFR.

### Histology and immunohistochemistry

Formaline fixed paraffin embedded kidney sections were evaluated after Masson’s trichrome staining. The degree of glomerulosclerosis and tubulointerstitial damage was determined blinded on a semiquantitative scale as previously described [23].

Briefly, glomerulosclerosis index (GSI) of each animal was determined with a light microscope at 400x magnification from the arithmetic mean of 100 evaluated glomeruli. The tubulointerstitial damage index (TDI) scores were evaluated at 100x magnification as follows: score 0: no change; score 1–5 depending on the criteria have been met in the given field of view: tubular dilatation, tubular atrophy, hyalin in tubular lumen, interstitial infiltration of mononuclear cells, interstitial fibrosis.

Immunohistochemical staining of paraffin embedded sections was performed using avidin-biotin method as previously described [23] using citrate buffer pH 6.0 for heat induced antigen retrieval. The primary antibodies were rabbit polyclonal anti-fibronectin at 1:1000 (Sigma-Aldrich, Budapest), and rabbit polyclonal anti-EGR-1 at 1:500 (Cell Signaling, USA).

Immunostaining reactivitiy scores were evaluated in a blinded manner at 400x magnification using a semi-quantitative scoring method: score 0: no staining; score 1: light staining; score 2: moderate staining; score 3: strong staining; score 4: very strong staining. Renal EGR-1 expression was assessed by counting the amount of EGR-1 positive nuclei at 400x magnification (high power field, HPF) and expressed as positive cells / HPF.

### Gene expression analysis

RNA was isolated from total kidney homogenates (20–30 mg) by classical phenol-chloroform extraction (Trizol, Thermo Fisher) according to the manufacturer’s protocol. The RNA concentration was measured photometrically with NanoDrop (Thermo Fisher). Reverse transcription of 1 μg RNA was performed using High Capacity cDNA Reverse Transcription kit (Applied Biosystems, Forster City, CA, USA). Each PCR reaction was measured by BioRad CFX96 device (BioRad, USA), using Bioline SensiFast SYBR Green PCR Master Mix (Bioline, Germany) The specificity and effectivity of PCR reactions were verified by melting curve analysis. Each sample was measured in duplicate and normalized for ribosomal 18S RNA expression using the *2*^*-*Δ*Ct*^ formula. Primer sequences were as follows: *mActa2 forward:* ATAACCCTTCAGCGTTCAGC; *mActa2 reverse:* ACATAGCTGGAGCAGCGTC; *mCol1a1 forward:* CATAAAGGGTCATCGTGGCT; *mCol1a1 reverse:* TTGAGTCCGTCTTTGCCAG; *mCol3a1 forward:* TGGAAAAGATGGAACAAGTGG; *mCol3a1 reverse:* CCAGACTTTTCACCTCCAAC; *mCtgf forward:* CCCGAGTTACCAATGACAATAC; *mCtgf reverse:* CTTAGCCCTGTATGTCTTCAC; *mFos forward:* TTTCAACGCCGACTACGAGG; *mFos reverse:* GCGCAAAAGTCCTGTGTGTT; *mJun forward:* GCACATCACCACTACACCGA; *mJun reverse:* GGGAAGCGTGTTCTGGCTAT; *mLcn2 forward:* ACGTCACTTCCATCCTCGTC; *mLcn2 reverse:* CCTGGAGCTTGGAACGAATG; *mMmp9 forward:* TGGATAAGGAGTTCTCTGGTG; *mMmp9 reverse:* CCACCTTGTTCACCTCATTTT; *mTgfb1 forward:* CACCATCCATGACATGAACC; *mTgfb1 reverse:* TCATGTTGGACAACTGCTCC; *mTimp1 forward:* CACCAGAGCAGATACCATG; *mTimp1 reverse:* GTGGTCTCGTTGATTTCTGG. The individual gene expression levels in each experimental group were normalized to a calibrator (control sample) in order to exclude unwanted sources of variation, and each gene expression values are expressed as fold expression relative to this control sample.

### Statistics

Experimental data are presented as mean ± SD and statistical analysis was performed using SPSS 10 for Windows (SPSS Inc). The data were analyzed using Kruskal-Wallis test followed by Dunn’s multiple comparison test. The level of significance was set to *p* < 0.05. The data and statistical analysis comply with the recommendations on experimental design and analysis in pharmacology [24].

## Results

### Murine plasma TGF-β_1_ levels, body weight and blood pressure

Both wild type control mice and TGF-β_1_ transgenic animals were evaluated for the absence or presence of the transgene. Control mice did not show the amplification of transgene, while transgenic mice showed the porcine TGF-β_1_ PCR product (Additional file 1: Figure S1). Accordingly, the plasma TGF-β_1_ levels of transgenic mice was significantly elevated as compared to control mice (Additional file 1: Figure S1).

**Fig. 1:**
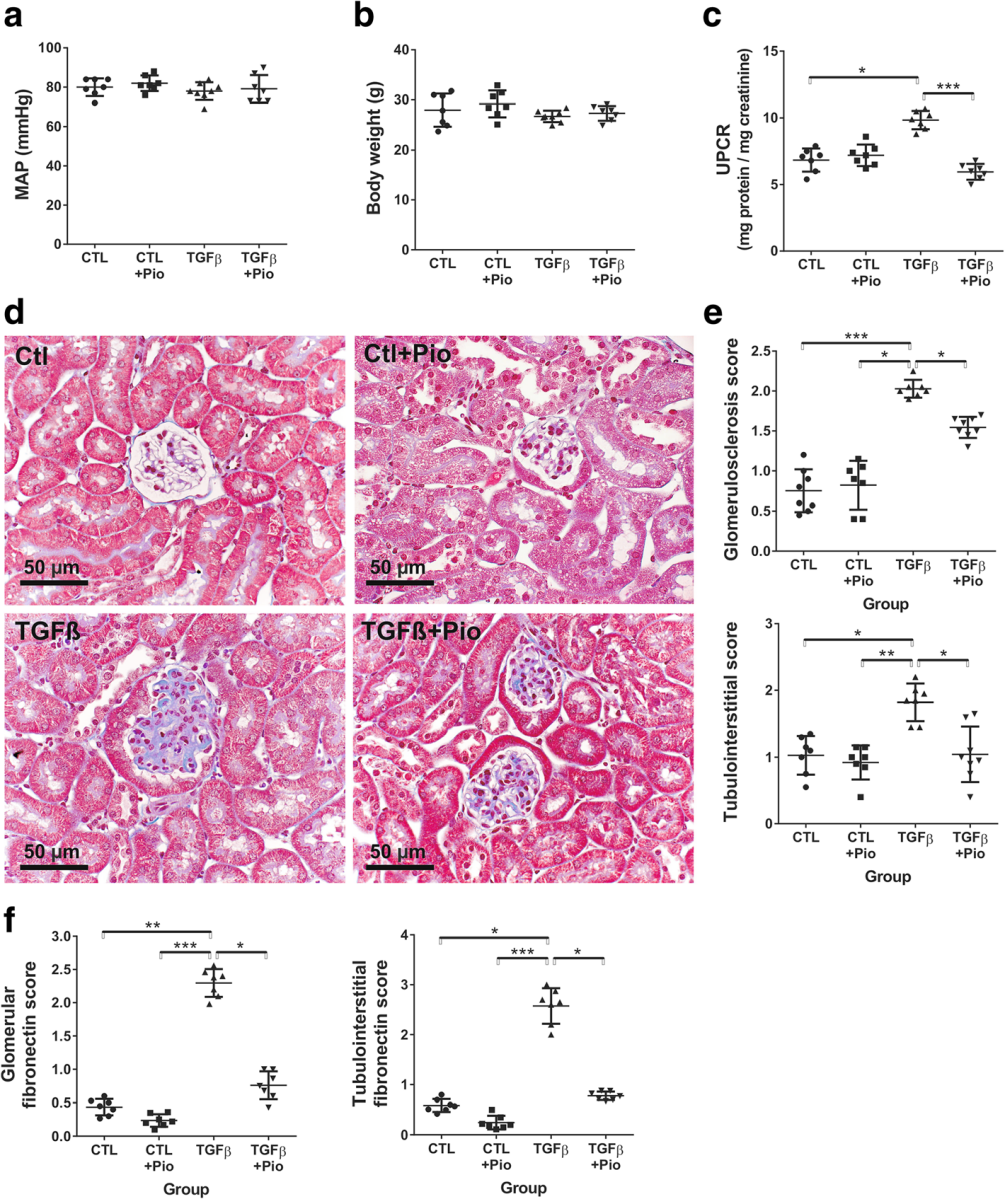
The values of mean arterial pressure, body weights, urinary protein/creatinine ratio and renal histology. Both mean arterial blood pressure (MAP, **a**) and body weight of control and transgenic mice (**b**) was similar in all groups regardless of treatment. Urinary protein/creatinine ratio (UPCR) was elevated in untreated TGF-β_1_ transgenic mice (**c**), but was significantly ameliorated by pioglitazone treatment. Renal histology depicted significant glomerulosclerosis and tubulointerstitial damage in untreated TGF-β_1_ mice (**d** and **e**) that were reduced by pioglitazone (Masson’s trichrome staining, 400X magnification, bar represents 50 μm). Fibronectin immunostaining was markedly stronger in both glomeruli and tubulointerstitium of untreated TGF-β_1_ mice (**f**), but reduced to control levels in pioglitazone treated mice (*n* = 7/group, *:*p* < 0.05, **:*p* < 0.01, ***:*p* < 0.001, Kruskal-Wallis test)

Both control and TGF-β_1_ transgenic mice had similar body weight regardless of the treatment (Fig. 1b), and the mean arterial blood pressure was also similar in all groups (Fig. 1a).

### Pioglitazone ameliorates TGF-β_1_ induced proteinuria, glomerular and tubular injury

Urinary protein/creatinine ratio (UPCR) was similar in both the pioglitazone treated and non-treated controls. In contrast, UPCR was elevated by 40% in untreated TGF-β_1_ transgenic mice, as compared to controls, but was significantly reduced to near normal levels in pioglitazone treated transgenic mice (Fig. 1c.).

Kidneys of control animals depicted normal histology without signs of glomerular or tubular damage, independent of the therapy (Fig. 1d and e). In contrast, and consistent with the UPCR results, untreated TGF-β_1_ transgenic kidneys depicted extensive glomerulosclerosis and significant tubulointerstitial damage, accompanied by marked renal fibronectin immunoreactivity (Fig. 1f). Pioglitazone treatment reduced the TGF-β_1_ induced glomerulosclerosis and tubulointerstitial damage by 30% and by 50%, respectively (Fig. 1d and e), and normalized fibronectin expression (Fig. 1f).

**Fig. 2:**
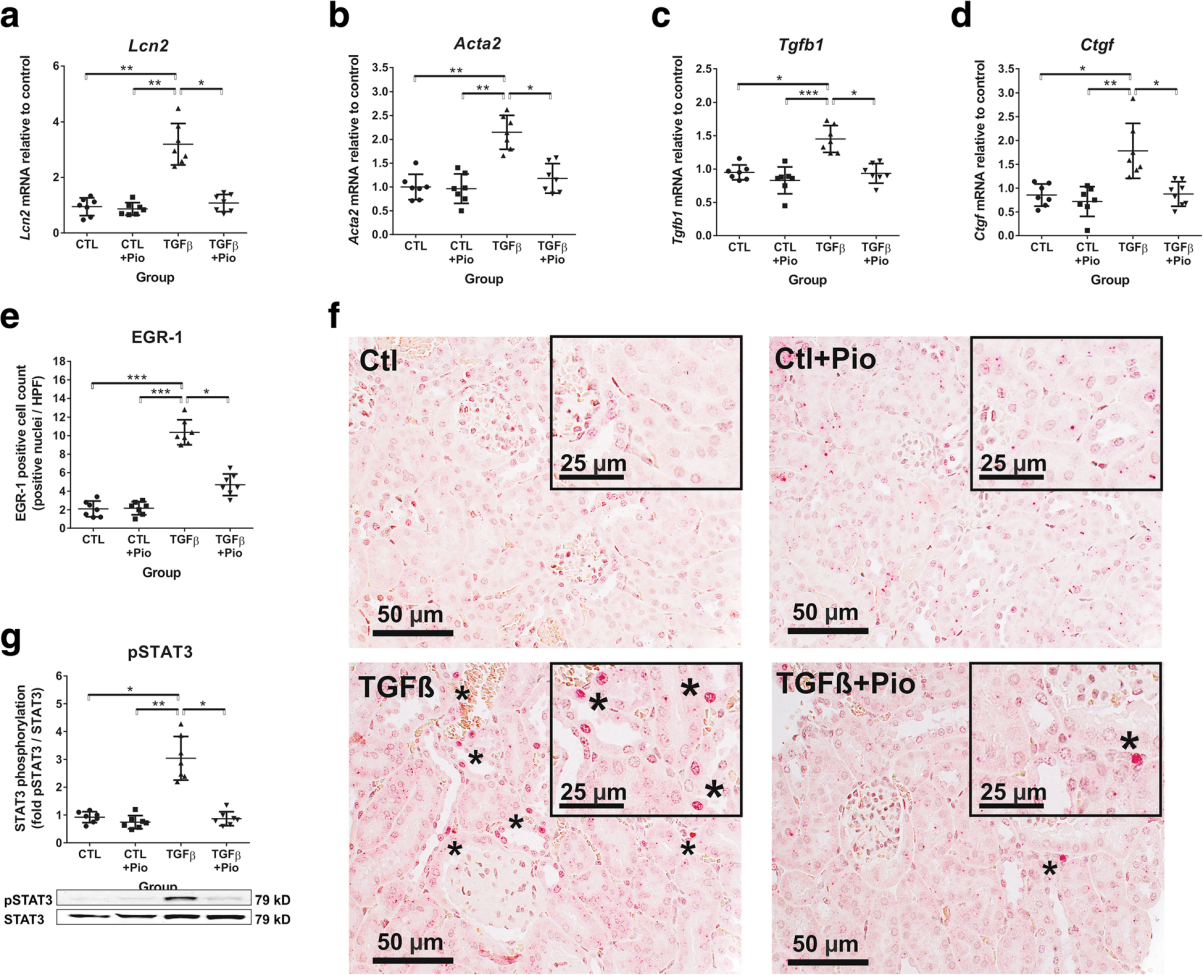
Gene and protein expression of fibrosis related molecules. As compared to control mice, the renal mRNA expression of lipocalin-2 *(Lcn2)* (**a**), alfa-smooth muscle actin *(Acta2)* (**b**), TGF-β_1_ *(Tgfb1)* (**c**) and CTGF *(Ctgf)* (**d**) were elevated in untreated TGF-β_1_ transgenic mice, but reduced to control levels by pioglitazone treatment. The kidneys of untreated TGF-β_1_ mice depicted significantly more early growth response factor-1 (EGR-1) positive nuclei (**e** and **f**, see asterisks), as compared to controls or pioglitazone treated TGF-β_1_ mice (400x magnification, bar represents 50 μm; insets: 630x magnification). The strong fibrotic response in TGF-β_1_ kidneys was associated with significant STAT3 phosphorylation (**g**). Kruskal-Wallis test (*n* = 7/group), *:*p* < 0.05, **:*p* < 0.01, ***:*p* < 0.001

In line with our histological findings, mRNA expression levels of Lipocalin-2 (*Lcn2*, a sensitive biomarker of tubular damage [25]) were similar in the pioglitazone treated and non-treated controls. In contrast, *Lcn2* expression was significantly elevated in untreated TGF-β_1_ transgenic mice but reduced to near normal levels in pioglitazone treated transgenic mice (Fig. 2a).

### Pioglitazone improves TGF-β_1_ induced renal fibrosis and transcription factor expression

During renal fibrosis, myofibroblasts express α-smooth muscle actin *(Acta2)*, and TGF-β_1_ transgenic kidneys overexpressed *Acta2* by 2-fold as compared to controls. Pioglitazone treatment was able to reduce *Acta2* expression to near control levels (Fig. 2b).

Among the profibrotic growth factors, renal expression of both TGF-β_1_ *(Tgfb1)* and CTGF *(Ctgf)* mRNA were significantly reduced by pioglitazone treatment (Fig. 2c and d). Similarly, we found strong EGR-1 immunoreactivity in TGF-β_1_ transgenic kidneys that was attenuated by pioglitazone treatment (Fig. 2e and f).

We observed marked STAT3 phosphorylation in the kidneys of untreated TGF-β_1_ transgenic mice, as compared to control groups, that was normalized by pioglitazone administration (Fig. 2g).

**Fig. 3:**
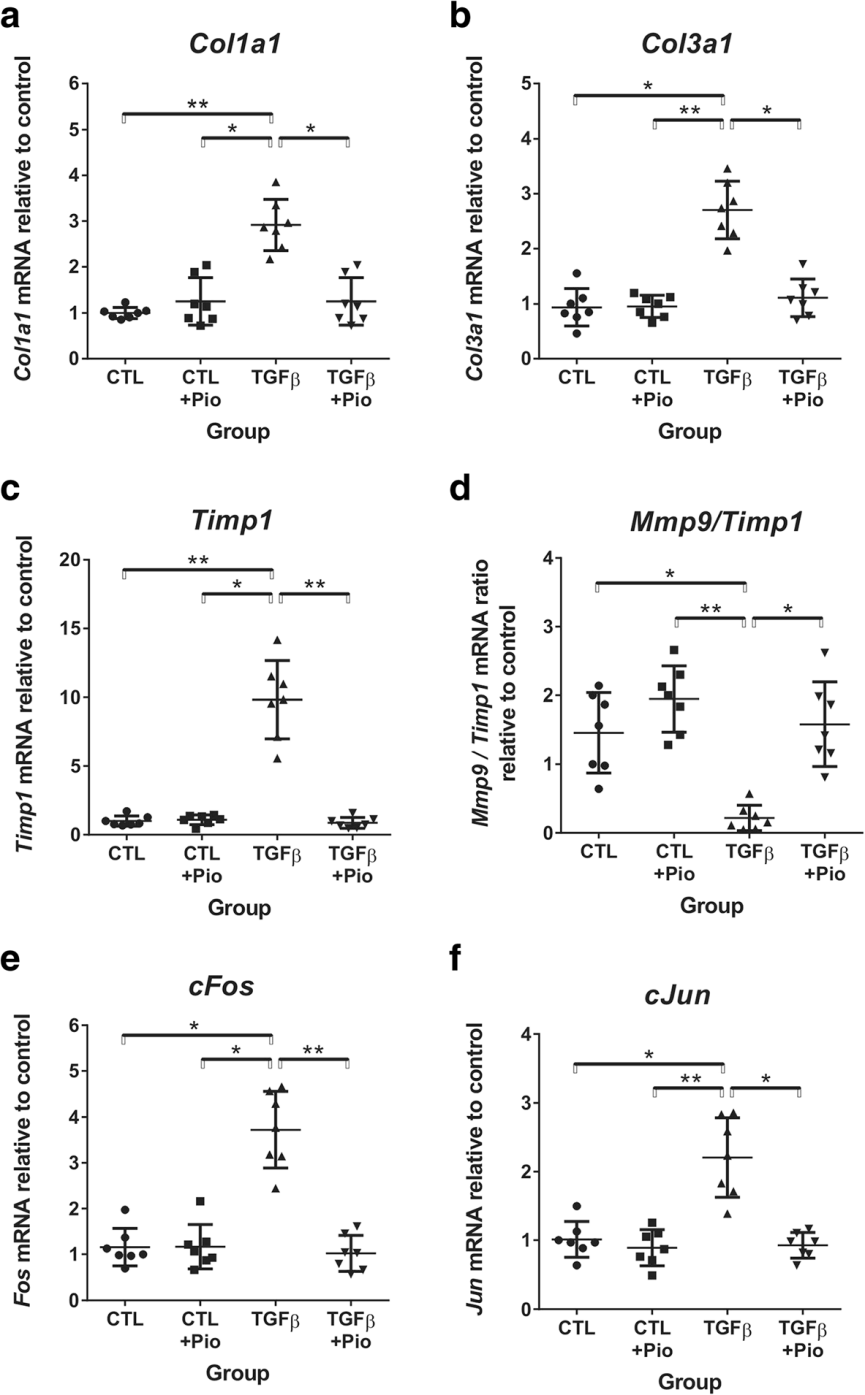
Gene expression of extracellular matrix components and activator-protein 1 components. The mRNA expression of type I *(Col1a1)* and type III *(Col3a1)* collagens were significantly elevated in TGF-β_1_ kidneys (**a**, **b**), accompanied by marked TIMP-1 mRNA *(Timp1)* overexpression (**c**) with reduced MMP-9/TIMP-1 ratio (**d**) which were attenuated by pioglitazone. Kidneys of untreated TGF-β_1_ mice also had elevated *cFos* and *cJun* expression, normalized by pioglitazone treatment (**e**, **f**). Kruskal-Wallis test (*n* = 7/group), *:*p* < 0.05, **:*p* < 0.01

In accordance with the pro-fibrotic effect of circulating TGF-β_1_ in transgenic mice, the renal expression of type I *(Col1a1)* and type III collagens *(Col3a1)* were 3-fold higher as compared to controls, but significantly attenuated by pioglitazone treatment (Fig. 3a and b). The increased production of extracellular matrix components was accompanied by a 10-fold overexpression of TIMP-1 mRNA *(Timp1)* in untreated transgenic mice, accompanied by strikingly reduced renal MMP-9/TIMP-1 ratio, as compared to controls (Fig. 2c and d). Pioglitazone dampened *Timp1* overexpression in transgenic mice and normalized the MMP-9/TIMP-1 ratio without any effect on the control groups (Fig. 2c and d).

The observed *Timp1* overexpression in TGF-β_1_ transgenic kidneys was accompanied by 4-fold and 2-fold increase in mRNA expression of the AP-1 components *cFos* and *cJun*, respectively (Fig. 2e and f), all of which were completely blocked (to control levels) by oral pioglitazone treatment (Fig. 2e and f).

## Discussion

In the present study, we examined the effect of PPARγ activation on the transcriptional regulation of renal fibrosis in TGF-β_1_ transgenic mice. To the best of our knowledge, this is the first report demonstrating that the PPARγ agonist pioglitazone reduced kidney fibrosis in association with dampened renal EGR-1 and TIMP-1 expression.

PPARγ agonists (e.g. pioglitazone) have been widely used in the treatment of pre-diabetes and diabetes mellitus [18, 26]. However, its effects on renal fibrosis in animal models of renal ischemia reperfusion injury, autosomal dominant polycystic kidney disease (ADPKD) or nondiabetic chronic kidney diseases have been ambigous [19, 21], and yet, the possible role of transcription factors have not been elucidated.

In our study, neither the several fold increased plasma TGF-β_1_ concentration, nor pioglitazone treatment had any influence on the mean arterial blood pressure of mice. This is in contrast to some reports on PPARγ agonists having antihypertensive effects, caused by increased endothelial NO levels and inhibition of ANG II [27]. However, several other studies did not find changes in systemic blood pressure with PPARγ agonist treatment (rosiglitazone) in wild type mice on regular and high fat diet [28]. Our study does support the beneficial effect of PPARγ agonists on reducing proteinuria, as previously shown in hypertensive and diabetes animal models [20], as well as T2DM patients [27]. Accordingly, pioglitazone significantly ameliorated both glomerular and tubulointerstitial fibrotic changes in the TGF-β_1_ transgenic mice, associated with decreased expression of lipocalin-2, a sensitive biomarker of tubular damage [25]). Along with these findings, mRNA expression of fibronectin, type I and type III collagens, and TGF-β_1_ were reduced, all known hallmarks of renal fibrosis [29–31]. Increased renal TGF-β_1_ level promotes endothelial-mesenchymal transition (EMT) and progressive renal interstitial fibrosis [32]. CTGF, as one of the downstream TGF-β_1_ mediators, participates in fibroblast proliferation and ECM production [19]. In our study, oral pioglitazone administration inhibited the TGF-β_1_-driven CTGF mRNA expression. The anti-fibrotic and antiproliferative effect of pioglitazone in our studies is supported by animal models of 5/6 nephrectomy [33], passive Heymann nephritis [34], acute mesangial proliferative glomerulonephritis, UUO [20] and ischemia reperfusion injury [35]. We have recently demonstrated that pioglitazone attenuates TGF-β_1_ induced pulmonary arterial hypertension and remodeling in the TGF-β_1_ overexpressing mouse, via decreasing TGF-β_1_, CTGF and α-smooth muscle actin (Acta2) expression in pulmonary arterial SMC [16]. Furthermore, PPARγ agonists might directly inhibit TGF-β_1_ expression and reduce interstitial myofibroblast accumulation [20]. In our current study, the reduced α-smooth muscle actin and renal TGF-β_1_ expressions support the proposed inhibitory effect of PPARγ agonists on myofibroblast activation and ECM production in fibrotic kidneys [36].

TGF-β_1_ not only induces the synthesis of ECM components but also reduces matrix degradation via tissue inhibitors of metalloproteinases (TIMPs) [37]. Untreated TGF-β_1_ transgenic mice in our study had disrupted renal MMP/TIMP balance that, intriguingly, was normalized by pioglitazone treatment. The latter could be, on one hand, a direct consequence of the reduced renal TGF-β_1_ expression. On the other hand, pioglitazone might have indirect effect on the MMP/TIMP balance via transcriptional modulation. As TIMP-1 transcription is partly regulated by the activator protein-1 (AP-1) complex, we investigated the expression of the AP-1 components cFos and cJun [4], that were significantly increased in untreated TGF-β_1_ transgenic kidneys. Consistent with our proposal, both cFos and cJun expression was normalized by pioglitazone treatment. Our findings not only support the regulatory importance of AP-1 transcription complex, but also show that PPARγ counteracts AP-1 activation.

Other transcription factors, such as the immediate early gene EGR-1, might be induced by a variety of fibrogenic stimuli, directly stimulating collagen production, matrix accumulation and myofibroblast differentiation [6, 7]. We found boosted EGR-1 expression in untreated TGF-β_1_ transgenic kidneys that was attenuated by pioglitazone treatment. Although the relationship of EGR-1 and PPARγ in the kidney has not been investigated yet, the PPARγ agonist rosiglitazone decreased skin fibrosis and EGR-1 levels in a mouse model of scleroderma [38]. This latter finding supports our results and implicates that EGR-1 is a potential anti-fibrotic target of activated PPARγ.

In addition, activation of the JAK/STAT pathways may be responsible for the increased myofibroblast transdifferentiation in the injured kidney [39]. STAT3 can induce TGF-β_1_ expression [15], and STAT3 inhibition was reported to suppress tubulointerstitial fibrosis in UUO model [40]. Furthermore, we have recently demonstrated in human pulmonary arterial smooth muscle cells that TGF-β_1_ induces STAT3 phosphorylation, and this STAT3 activation is inhibited by pioglitazone [16]. Our present study shows an additional potential interplay of STAT3 and PPARγ in renal fibrosis, as STAT3 was activated in kidneys of untreated TGF-β_1_ transgenic mice, but STAT3 activation was absent in kidneys of TGF-β_1_ transgenic mice chronically treated with pioglitazone.

**Fig. 4:**
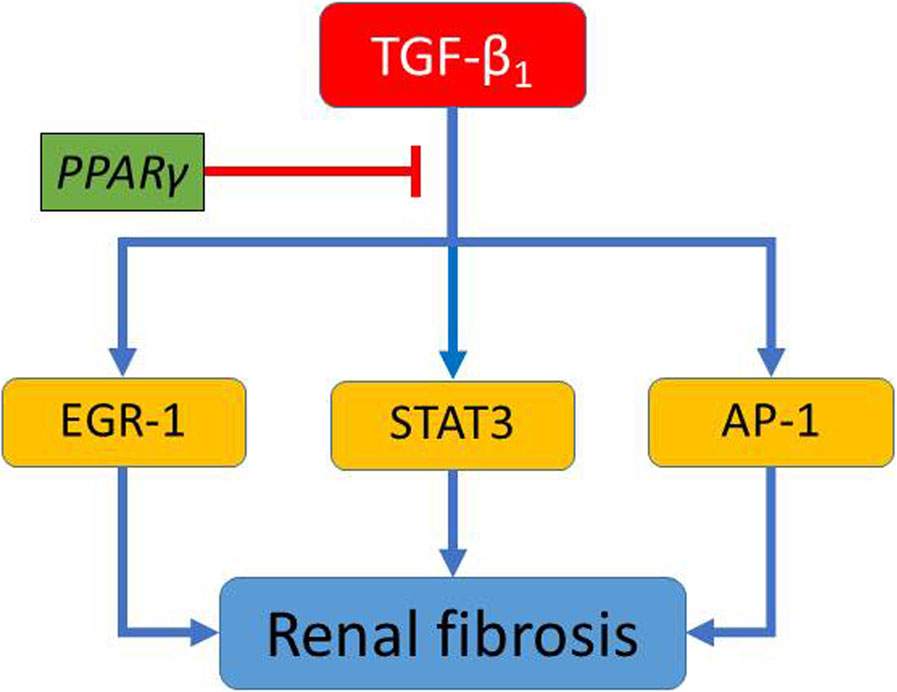
Proposed novel mechanism how the PPARγ agonist pioglitazone attenuates TGF-β_1_ induced renal fibrosis. Our study shows that PPARγ agonist pioglitazone repressed the transcription factors EGR-1, STAT3, and AP-1 (cFos, cJun), all of which are important signaling mediators in the pathomechanisms of TGF-β_1_ driven renal fibrosis

Based on our study, we postulate that PPARγ agonist pioglitazone exerts its antifibrotic effect in the kidney by repressing STAT3 activation as well as decreasing the expression of EGR-1 and AP-1 components (cFos, cJun) (Fig. 4).

## Conclusions

To the best of our knowledge, this is the first study to show that oral administration of PPARγ agonist pioglitazone in fibrosis prone TGF-β_1_ transgenic mice antagonized the profibrotic effects of TGF-β_1_ by repressing EGR-1, STAT3 and AP-1. In addition, pioglitazone normalized the MMP/TIMP imbalance in the kidneys without changing the systemic blood pressure. Our observations and previous reports suggest that PPARγ agonists might be effective in the future treatment of chronic kidney disease patients, especially in the context of heightened TGF-β_1_ signaling.

As a limitation, we did not test the renal effect of direct STAT3 or EGR-1 inhibition in our model, eg. by using in vivo gene silencing approach. However, such expansion of our study would have increased the amount of animals needed, and would introduce a significant bias due to the intrarenal delivery problems of gene silencing oligonucleotides.

## Additional file

Additional file 1: **Figure S1.** (a) Representative picture of genotyping the TGF-β transgenic mice (samples 3,4 and 6 are transgenic, showing the 370 bp PCR product of the transgene; samples 1,2 and 5 are wild type controls). (b) Levels of circulating TGF-β1 in wild type B6 control mice (CTL) and transgenic mice (TGFβ) at the beginning of the study clearly shows that transgenic mice had 3-fold elevated plasma TGF-β1 levels (*n* = 14/group, *p* < 0.001, Mann-Whitney test). (PDF 209 kb)

## Data Availability

The datasets used and/or analysed during the current study are available from the corresponding author on reasonable request.

## Abbreviations

Acta2: Gene of alfa-smooth muscle actin
AP-1: Activator protein-1
Col1a1: Gene of type I collagen alfa chain
Col3a1: Gene of type III collagen alfa chain
CTGF: Connective tissue growth factor
ECM: Extracellular matrix
EGR-1: Early growth response factor-1
ESRD: End-stage renal disease
Lcn2: Gene for lipocalin-2
MAP: Mean arterial blood pressure
MMP-9: Matrix metalloprotease-9
PPARγ: Peroxisome proliferator-activated receptor-γ
pSTAT3: phosphorylated Signal transducers and activators of transcription-3
STAT3: Signal transducers and activators of transcription-3
Tgfb1: Gene of transforming growth factor-ß1
TGF-ß1: Transforming growth factor-ß1
TIMP-1: Tissue inhibitor of matrix metalloprotease-1
UPCR: Urinary protein / creatinine ratio
