# Supplementary material for: The PPARγ agonist pioglitazone prevents TGF-β induced renal fibrosis by repressing EGR-1 and STAT3
Source: BMC Nephrol. 2019 Jul 5;20:245. doi: 10.1186/s12882-019-1431-x (PMC6610924; doi:10.1186/s12882-019-1431-x)
Supplement: Supplementary file 1 — Figure S1. (a) Representative picture of genotyping the TGF-β transgenic mice (samples 3,4 and 6 are transgenic, showing the 370 bp PCR product of the transgene; samples 1,2 and 5 are wild type controls). (b) Levels of circulating TGF-β1 in wild type B6 control mice (CTL) and transgenic mice (TGFβ) at the beginning of the study clearly shows that transgenic mice had 3-fold elevated plasma TGF-β1 levels (n = 14/group, p < 0.001, Mann-Whitney test). (PDF 209 kb) [file 12882_2019_1431_MOESM1_ESM.pdf]

Title: Pioglitazone prevents TGF-β induced renal fibrosis by repressing EGR-1 and STAT3

Authors: Ágnes Németh, Miklós M. Mózes, Laurent Calvier, Georg Hansmann, Gábor Kökény

Supplementary material

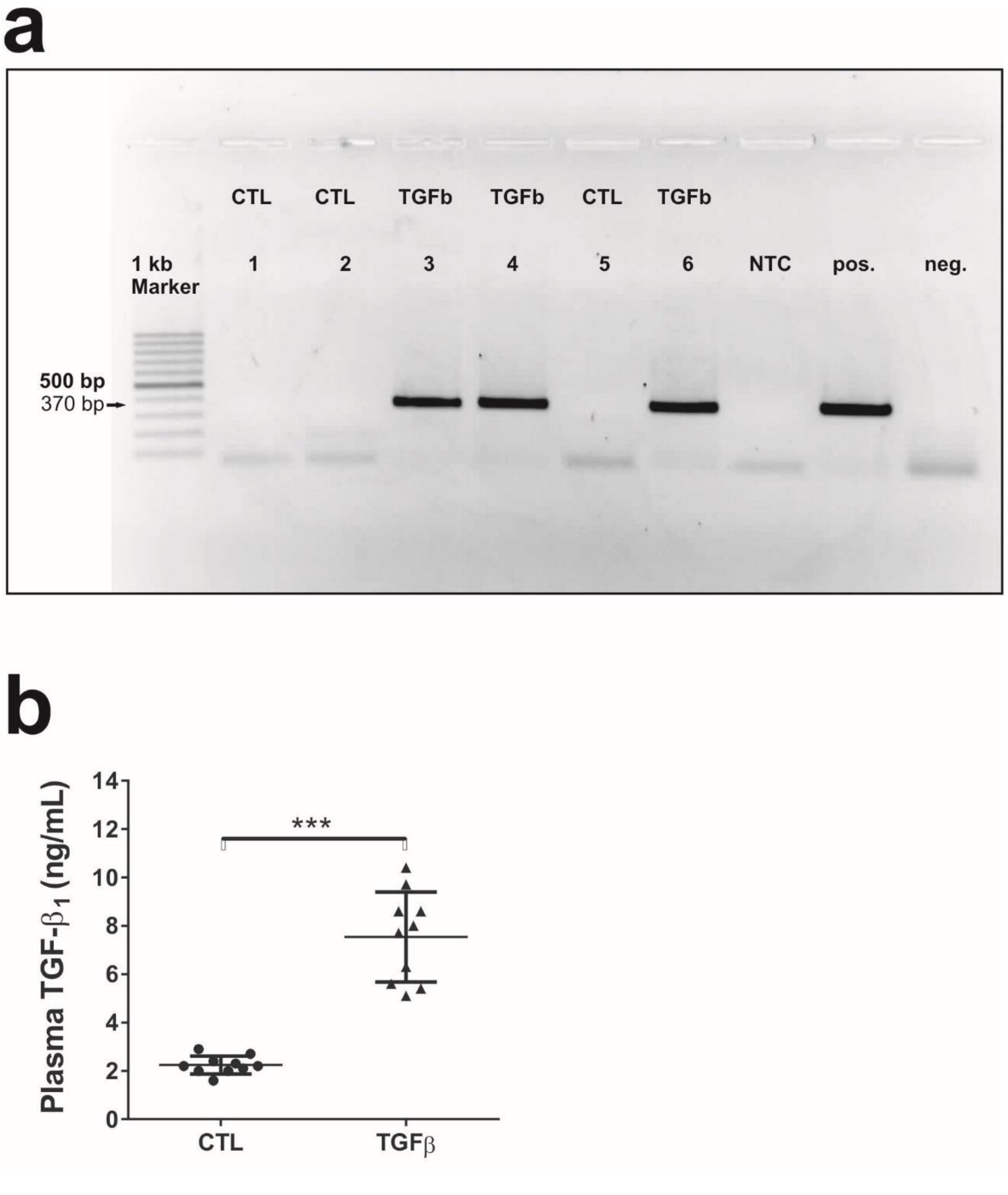

Supplementary Figure 1. (a) Representative picture of genotyping the TGF-β transgenic mice (samples 3,4 and 6 are transgenic, showing the 370 bp PCR product of the transgene; samples 1,2 and 5 are wild type controls). (b) Levels of circulating TGF-β1 in wild type B6 control mice (CTL) and transgenic mice (TGFβ) at the beginning of the study clearly shows that transgenic mice had 3-fold elevated plasma TGF-β1 levels (n=14/group, p<0.001, Mann-Whitney test).
